# Xanthine oxidase inhibitory activity of a new isocoumarin obtained from *Marantodes pumilum* var. *pumila* leaves

**DOI:** 10.1186/s12906-020-03119-8

**Published:** 2020-10-27

**Authors:** Nor-Ashila Aladdin, Khairana Husain, Juriyati Jalil, Carla Wulandari Sabandar, Jamia Azdina Jamal

**Affiliations:** 1 Drug and Herbal Research Centre, Faculty of Pharmacy, Universiti Kebangsaan Malaysia, Kuala Lumpur, Malaysia; 2 Department of Pharmacy, Faculty of Science and Technology, Universitas Sembilanbelas November Kolaka, Kolaka, Indonesia

**Keywords:** *Marantodes pumilum* var. *pumila*, Xanthine oxidase inhibitor, Isocoumarin, Hyperuricemia, Gout

## Abstract

**Background:**

In traditional Malay medicine, *Marantodes pumilum* (Blume) Kuntze (family Primulaceae) is commonly used by women to treat parturition, flatulence, dysentery, dysmenorrhea, gonorrhea, and bone diseases. Preliminary screening of some Primulaceae species showed that they possess xanthine oxidase inhibitory activity. Thus, this study aimed to investigate the xanthine oxidase inhibitory activity of three varieties of *M. pumilum* and their phytochemical compounds.

**Method:**

Dichloromethane, methanol, and water extracts of the leaves and roots of *M. pumilum* var. *alata*, *M. pumilum* var. *pumila,* and *M. pumilum* var. *lanceolata* were tested using an in vitro xanthine oxidase inhibitory assay. Bioassay-guided fractionation and isolation were carried out on the most active extract using chromatographic techniques. The structures of the isolated compounds were determined using spectroscopic techniques.

**Results:**

The most active dichloromethane extract of *M. pumilum* var. *pumila* leaves (IC_50_ = 161.6 μg/mL) yielded one new compound, 3,7-dihydroxy-5-methoxy-4,8-dimethyl-isocoumarin (**1**), and five known compounds, viz. ardisiaquinone A (**2**), maesanin (**3**), stigmasterol (**4**), tetracosane (**5**), and margaric acid (**6**). The new compound was found to be the most active xanthine oxidase inhibitor with an IC_50_ value of 0.66 ± 0.01 μg/mL, which was not significantly different (*p* > 0.05) from that of the positive control, allopurinol (IC_50_ = 0.24 ± 0.00 μg/mL).

**Conclusion:**

This study suggests that the new compound 3,7-dihydroxy-5-methoxy-4,8-dimethyl-isocoumarin (**1**), which was isolated from the dichloromethane extract of *M. pumilum* var. *pumila* leaves, could be a potential xanthine oxidase inhibitor.

## Background

*Marantodes pumilum* (Blume) Kuntze belongs to the Primulaceae family [1]. It was previously known as *Labisia pumila* (Blume) Fern.-Vill. from the Myrsinaceae family [2]. The taxonomic characteristics of eight varieties of *M. pumilum* have been described [3], and three of the varieties (var. *alata* Scheff., var. *pumila,* and var. *lanceolata* (Scheff.) Mez) are commonly used in Malaysia [2]. The close resemblance of var. *alata* and var. *pumila* leaves has made macromorphological identification quite difficult, as the leaf laminas of both varieties are either narrowly or broadly elliptic or ovate with 10–30 × 1.3–11 cm dimensions [3]. However, their petioles differ. The petiole of var. *alata* is 5–12 cm long and broadly winged (3–5 mm wide), whereas that of var. *pumila* is 4–15 cm long and slightly winged. Nonetheless, to differentiate them based on characteristic anatomical features and chemical profiling, a pharmacognostical study of these varieties was performed using microscopic, high-performance thin layer chromatography (HPTLC), high performance liquid chromatography (HPLC), and attenuated total reflectance-Fourier transform infrared spectroscopy (ATR-FTIR) techniques [4].

In traditional Malay medicine, *M. pumilum* decoction is popularly used among women to induce and facilitate labor, delay fertility, and regain vitality, as well as to treat flatulence, dysentery, dysmenorrhea, gonorrhea, and bone diseases [5–7]. Men of several ethnic groups in the Sarawak state of Malaysia also consume the plant to maintain and increase stamina [8]. Additionally, the plant has been increasingly used as a supplement and beverage among the public for general health maintenance [9]. Previous scientific studies have reported the activities of *M. pumilum*, including antioxidant [10], xanthine oxidase inhibition [11, 12], antimicrobial [13], anti-inflammatory [14], uterotonic effect [15], phytoestrogenicity [16], anti-obesity [17], anti-aging [18], and anti-carcinogenic [19]. Its phytochemical compositions such as triterpenoid saponins, alk(en) ylresorcinols, benzoquinone derivatives, fatty acids, flavonoids, and phenolics, have been documented [13, 20–23].

Xanthine oxidase (XO) catalyzes the oxidation of hypoxanthine to xanthine and xanthine to uric acid [24]. It plays a major role during the last step of purine nucleotide metabolism in humans, and serves as an important biological source of oxygen-derived free radicals. Free radicals can contribute to the oxidative damage to living tissues, which are involved in many pathological processes and various ischemic tissues, vascular injuries, and inflammation [25, 26]. Xanthine oxidase is primarily distributed in the liver and intestine [27]. In humans, overproduction of xanthine oxidase elevates the blood stream uric acid concentration and leads to hyperuricemia [28]. Uric acid deposition begins when uric acid dissolves in the blood and forms urate monohydrate crystals in the joints and kidneys, leading to painful inflammation. Uric acid has been identified as a marker for gout and several metabolic and hemodynamic abnormalities [25, 29, 30]. Synthetic xanthine oxidase inhibitors such as allopurinol, febuxostat, and phenylpyrazol derivative Y-700, have been widely used to treat hyperuricemia and gout [27], but may have side effects. The extensively prescribed allopurinol has been reported to cause Stevens-Johnson syndrome, toxic epidermal necrolysis, hepatic disorders, and renal dysfunction [31]. Therefore, new alternatives such as medicinal plants, with fewer side effects, are desired [32, 33].

Phytochemical constituents such as phenolics, flavonoids, coumarins, lignans, triterpenoids, and alkaloids have been reported to inhibit xanthine oxidase [27, 34–36]. Esculetin, a hydroxycoumarin derivative, displayed strong xanthine oxidase inhibitory activity [37] and was proposed as an appropriate bioactive quality control marker for a traditional Chinese medicine formula used in the treatment of hyperuricemia [38]. The extract of *M. pumilum* was reported to alleviate hyperuricemia in vivo [39]. Thus, in this study, potential xanthine oxidase inhibitors were determined by evaluating the xanthine oxidase inhibitory activity of *M. pumilum* varieties and isolated compounds using an in vitro assay. The compound could be used as an analytical marker for quality control purposes of *M. pumilum*-containing herbal products intended for hyperuricemia or gouty conditions.

## Methods

### Materials and equipment

Microplates (96-well) used in the in vitro assay were obtained from Thermo Multiskan Go (Waltham, MA, USA). The following adsorbents were used: silica gel 60 (5–40 μm, cat. no. 1.07747) was used for vacuum liquid chromatography (VLC), silica gel 60 (40–63 μm, cat. no. 1.09385) and Sephadex LH-20 (GE Healthcare, Upsalla, Sweden) were used for column chromatography (CC), and silica gel 60 GF254 (0.25 mm, cat. no. 1.05554) was used for thin layer chromatography (TLC). The silica gels were obtained from Merck (Darmstadt, Germany).

For structural elucidation of the isolated compounds, ultraviolet (UV) spectra were recorded in ethanol using a Shimadzu UV1800 UV-Vis spectrophotometer (Shimadzu Corp., Kyoto, Japan), and infra-red (IR) spectra were obtained using a Spectrum 100 FTIR spectrophotometer (PerkinElmer, Inc., Waltham, MA, USA) with an ATR technique. One-dimensional proton (^1^H) and carbon (^13^C) and two-dimensional nuclear magnetic resonance (NMR) spectra were determined using a Bruker Avance III 600 MHz spectrometer (Bruker BioSpin, Karlsruhe, Germany), while high-resolution electrospray ionization mass spectrometry (HR-ESI-MS) and electron ionization mass spectrometry (EI-MS) spectra were obtained using an Ultimate 3000 system, MicrOTOF-Q II (Bruker Daltonics, Bremen, Germany).

### Chemicals and reagents

Analytical grade organic solvents, including dichloromethane (DCM), methanol (MeOH), chloroform (CHCl_3_), dimethyl sulfoxide (DMSO), hexane, ethyl acetate (EtOAc), toluene, acetone, and ethanol (EtOH), were purchased from Merck (Darmstadt, Germany). For the bioassay, allopurinol, xanthine, and xanthine oxidase (cow’s milk) were purchased from Sigma-Aldrich Chemicals (St. Louis, MO, USA), while dimethyl sulfoxide (DMSO), hydrochloric acid (HCl), sodium hydroxide (NaOH), and potassium dihydrogen phosphate (KH_2_PO_4_) were purchased from Merck (Darmstadt, Germany).

### Preparation of *M. pumilum* extracts

Three wild varieties of *M. pumilum* were collected from the Bujang Melaka Forest Reserve in Malaysia and authenticated by Mr. Sani Miran^†^, a botanist from the Herbarium of Universiti Kebangsaan Malaysia in Bangi (UKMB). The voucher specimens of var. *alata* (voucher number: UKMB 30006/SM 2622), var. *pumila* (UKMB 30007/SM s.n.), and var. *lanceolata* (UKMB 30008/SM s.n.) were deposited in the Herbarium of Universiti Kebangsaan Malaysia.

Leaves and roots (consisting of both stems and roots) of the fresh plants were separated and air-dried under shade. Following this, they were coarsely ground to obtain six powdered plant materials: var. *alata* leaves (0.2 kg) and roots (0.8 kg), var. *pumila* leaves (0.8 kg) and roots (2.0 kg), and var. *lanceolata* leaves (0.2 kg) and roots (0.5 kg). Within 1 week, each plant powder was successively macerated with dichloromethane in a powder-to-solvent ratio of 1:5, followed by methanol (ratio of 1:5). The methanol residue was refluxed with distilled water in a residue-to-solvent ratio of 1:13 for the leaves and 1:10 for the roots. The dichloromethane and methanol fluid extracts were vacuum-dried, and the water extracts were freeze-dried. This process resulted in eighteen dried extracts, which were stored in a refrigerator at 4 °C until further analyses.

### In vitro xanthine oxidase assay

The xanthine oxidase inhibitory assay was carried out using a previously reported method [40] with slight modifications. Initially, allopurinol (the positive control) and the dichloromethane and methanol extracts were dissolved in dimethyl sulfoxide (DMSO), and the water extracts were dissolved in distilled water. This was followed by dilution with potassium phosphate buffer (0.05 M, pH 7.5) to achieve the desired concentrations. Each test solution contained 0.5% DMSO. The assay was performed in triplicates in a 96-well microplate. The assay reaction mixture, which consisted of 130 μL of buffer, 10 μL of either test solution (400 μg/mL for extracts and 100 μg/mL for isolated compounds) or allopurinol (100 μg/mL), and 10 μL of xanthine oxidase (0.2 U/well), was incubated at 25 °C for 15 min. Then, 100 μL of substrate solution, xanthine (0.15 mM, pH 7.5), was added before further incubating at 25 °C for 10 min. The final assay mixture was spectrophotometrically measured at 295 nm. Xanthine oxidase inhibitory activity was expressed as the percentage of xanthine oxidase inhibition and calculated using the following formula:

$$ \% Xanthine\ Oxidase\ Inhibition=\left[\frac{\left(A-B\right)-\left(C-D\right)}{\left(A-B\right)}\right]\times 100 $$

Where A is the optical density without the test solution or allopurinol, B is the optical density of blank solution containing only potassium phosphate buffer (0.05 M, pH 7.5), C is the optical density of the test solution or allopurinol with the presence of xanthine oxidase, and D is the optical density of the test solution or allopurinol without xanthine oxidase. Test solutions with more than 50% xanthine oxidase inhibition were reassayed at concentrations of 25, 50, 100, 200, and 400 μg/mL for extracts, 0.39, 0.78, 1.56, 3.13, 6.25, 12.5, 25, 50 and 100 μg/mL for compound **1**, and 6.25, 12.5, 25, 50, and 100 μg/mL for compound **2**. Their half-maximal inhibitory concentration (IC_50_) values were determined from percentages of xanthine oxidase inhibition of the respective concentration range using GraphPad Prism 5 software (La Jolla, CA, USA) and compared with that of allopurinol (0.0064, 0.032, 0.16, 0.8, 4, 20, and 100 μg/mL).

### Isolation and structural elucidation of compounds from *M. pumilum* var. *pumila*

**Fig. 1:**
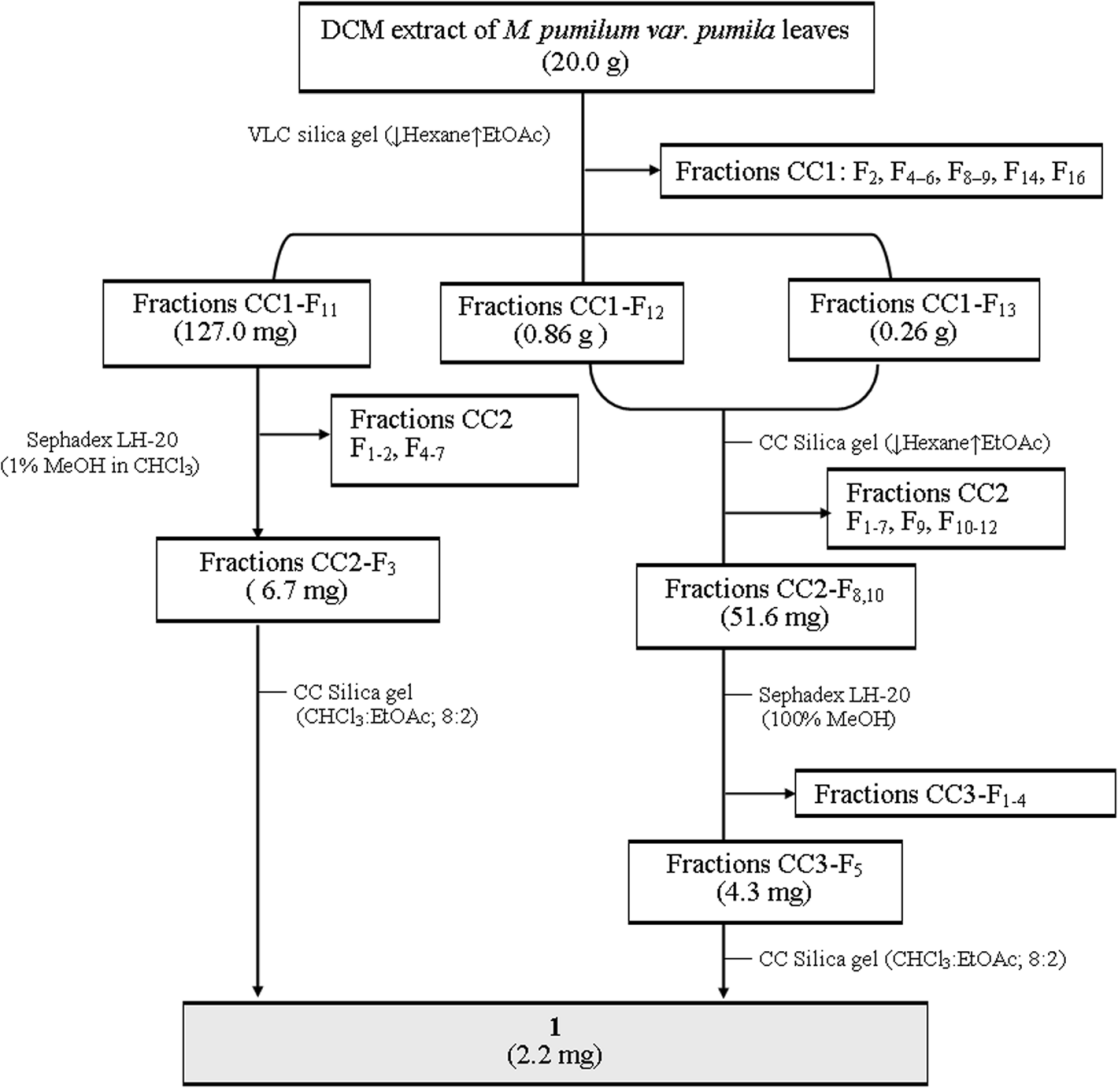
Flowchart of bioassay-guided isolation of compound **1** from dichloromethane extract of *Marantodes pumilum* var. *pumila* leaves

The screening assay revealed that the dichloromethane extract of *M. pumilum* var. *pumila* leaves was most active. The extract (20.0 g) was fractionated by vacuum liquid chromatography using silica gel and gradient elution with increasing polarity mobile phase, that is, 3 L of hexane-ethyl acetate (9:1, 8:2, 7:3, 6:4, 5:5, 3:7, 2:8, and 1:9) followed by 2 L of 100% ethyl acetate and 2 L of 100% methanol. Eluents (250 mL each) were collected and combined based on the similarity of TLC profiles to obtain 16 fractions (CC1: F_1–16_) (Fig. 1). The fractions were further fractionated using various chromatographic techniques with different solvent compositions to obtain six pure compounds.

#### 3,7-dihydroxy-5-methoxy-4,8-dimethyl-isocoumarin (1)

The fraction CC1-F_11_ (0.13 g) was fractionated using Sephadex LH-20 column chromatography (Φ 25 mm) with 1% methanol in chloroform to yield seven fractions that were assayed for xanthine oxidase inhibition. The third fraction was then eluted using silica gel column chromatography with chloroform-ethyl acetate (4:1) to obtain pure compound **1**. Compound **1** was also isolated from fractions CC1-F_12_ (0.86 mg) and CC1-F_13_ (0.26 mg) via several steps of bioassay-guided column chromatography and xanthine oxidase inhibition assay (Fig. 1). Compound **1** was obtained as a white amorphous solid (2.2 mg), and the data for its structural elucidation were as follows: TLC: R_f_ 0.3 (toluene-acetone, 9:1); UV (EtOH) λ_max_ nm (log ε): 275 (3.17); IR (ATR) ʋ_max_, cm^− 1^: 3256, 2926, 2952, 1734, 1659, 1607, 1464, 1383, 1311, and 1202; EI-MS *m/z*: 236 [M]^+^ (calculated for C_12_H_12_O_5_, 236.2207 g/mol); ^1^H-NMR (CDCl_3_, 600 MHz) δ_H_ (ppm): 2.45 (3H, *s,* H-11), 2.65 (3H, *s,* H-12), 3.89 (*s,* OCH_3_), and 6.91 (*s,* H-6); ^13^C-NMR (CDCl_3_, 150 MHz) δ_C_ (ppm): 162.7 (C-1), 157.1 (C-3), 139.9 (C-4), 110.2 (C-4a), 153.1 (C-5), 101.8 (C-6), 142.5 (C-7), 119.8 (C-8), 131.1 (C-8a), 18.2 (C-9), and 15.2 (C-10).

#### Ardisiaquinone A (2)

The fraction CC1-F_15_ (0.69 g) was fractionated using Sephadex LH-20 column chromatography with 1% methanol in chloroform to yield eight fractions. The third fraction was then eluted using silica gel column chromatography with chloroform-methanol (9:1) to obtain five fractions. The fourth fraction was triturated with hexane–methanol (1:1) to give compound **2**. Compound **2** was obtained as a yellow powder (5.0 mg), and the data for its structural elucidation were as follows: UV (EtOH) λ_max_ nm (log ε): 285 (3.15), and 206 (3.00); IR (ATR) ʋ_max_, cm^− 1^: 3346, 3342, 2923, 2954, 1633, 1595, 1463, 1311, 1202, 1078, and 838; HR-ESI-MS (+ve mode) *m/z*: 527.4275 [M-H]^+^ (calculated for C_30_H_40_O_8_, 528.4275 g/mol); ^1^H-NMR (CDCl_3_, 600 MHz) δ_H_ (ppm): 1.29 (16H, *m,* H-9-H-12, H-9′-H-12′, *overlapped*), 1.47 (4H, *m,* H-8, H-8′), 2.02 (4H, *m*, H-13, H-13′), 2.46 (4H, *m,* H-7, H-7′), 3.88 (*s*, OCH_3_), 5.35 (2H, *m,* H-14, H-14′), 5.86 (*d, J =* 3.0 Hz, H-6, H-6′), and 7.28 (*br s*, OH); ^13^C-NMR (CDCl_3_, 150 MHz) δ_C_ (ppm): 22.6 (C-7, C-7′), 27.2 (C-13, C-13′), 27.9 (C-8, C-8′), 29.2–29.7 (C-9-12, C-9′-12′), 129.7 (C-14, C-14′), 182.8 (C-1, C-1′), 151.5 (C-2, C-2′), 119.1 (C-3, C-3′), 181.7 (C-4, C-4′), 161.1 (C-5, C-5′), and 102.2 (C-6, C-6′).

#### Maesanin (3)

The fraction CC1-F_3_ (0.62 g) was fractionated using Sephadex LH-20 column chromatography with 1% methanol in chloroform to yield eight fractions. The fourth fraction was then eluted using silica gel column chromatography with chloroform-ethyl acetate (9:1), followed by trituration with hexane-methanol (1:1) to obtain compound **3**. Compound **3** was obtained as a yellow crystal (10.0 mg), and the data for its structural elucidation were as follows: UV (EtOH) λ_max_ nm (log ε): 285 (3.17) and 206 (2.97); IR (ATR) ʋ_max_, cm^− 1^: 3342, 2851, 2921, 1659, 1607, 1464, 1383, 1311, and 1200; HR-ESI-MS *m/z*: 363.5800 [M + H]^+^ (calculated for C_22_H_34_O_4_, 362.5800 g/mol); ^1^H-NMR (CD_3_OD, 600 MHz) δ_H_ (ppm): 0.93 (3H, *m,* H-15′), 1.29–1.34 (16H, *m,* H-3′-H-8′, H-13′-H-14′, *overlapped*), 1.45 (2H, *m,* H-2′), 2.05 (4H, *m,* H-9′, H-12′), 2.41 (2H, *t, J =* 7.8, H-1′), 3.85 (*s,* OCH_3_), 5.36 (2H, *m,* H-10′, H-11′), and 5.91 (*s,* H-6); ^13^C-NMR (CD_3_OD, 150 MHz) δ_C_ (ppm): 13.1 (C-15′), 21.9 (C-1′), 22.3–31.5 (C-2′-8′, C-13′-14′), 26.7 (C-9′, C-12′), 129.4 (C-10′, C-11′), 182.2 (C-1), 154.5 (C-2), 118.7 (C-3), 183.0 (C-4), 160.5 (C-5), 55.8 (OCH_3_), and 102.6 (C-6).

#### Stigmasterol (4)

The fraction CC1-F_7_ (2.0 g) was fractionated using Sephadex LH-20 column chromatography with 1% methanol in chloroform to yield ten fractions. The fifth fraction was purified by re-crystallization in methanol to yield compound **4**. Compound **4** was obtained as a white needle crystal (14.0 mg), and the data for its structural elucidation were as follows: mp 133–134°C; UV (EtOH) λ_max_ nm (log ε): 202 (3.76); IR (ATR) ʋ_max_, cm^− 1^: 3347, 2934, 2868, 1464, 1382, 1048, and 968; HR-ESI-MS *m/z*: 413.2666 [M + H]^+^ (calculated for C_29_H_48_O, 412.2470 g/mol); ^1^H-NMR (CDCl_3_, 600 MHz) δ_H_ (ppm): 0.72 (3H, *s*, H-18), 0.82 (3H, *d*, *J* = 6.6 Hz, H-27), 0.83 (3H, *t*, H-29), 0.87 (3H, *d*, *J* = 6.0 Hz, H-26), 0.95 (H-9), 1.01 (3H, *s*, H-19), 1.03 (H-14), 1.04 (3H, *d*, *J* = 6.6 Hz, H-21), 1.08 (H-15), 1.10 (H-1), 1.13 (H-17), 1.19 (H-12, H-28), 1.28 (H-16), 1.43 (H-28), 1.48 (H-8), 1.50 (H-7), 1.52 (H-2, H-11), 1.53 (H-25), 1.54 (H-24), 1.58 (H-15), 1.73 (H-16), 1.86 (H-2), 1.88 (H-1), 1.98 (H-7), 2.01 (H-12), 2.07 (H-20), 2.26 (H-4), 2.32 (H-4), 3.55 (H-3), 5.04 (*dd*, *J* = 15.3, 8.9 Hz, H-23), 5.17 (*dd*, *J* = 15.1, 8.7 Hz, H-22), and 5.37 (H-6); ^13^C-NMR (CDCl_3_, 150 MHz) δ_C_ (ppm): 11.9 (C-18), 12.3 (C-29), 19.0 (C-27), 19.4 (C-19), 21.1 (C-11, C-26), 23.1 (C-21), 24.3 (C-15), 26.0 (C-28), 29.1 (C-16), 31.7 (C-7, C-25), 31.9 (C-2, C-8), 36.2 (C-10), 37.3 (C-1), 39.7 (C-12), 40.6 (C-20), 42.3 (C-13), 45.8 (C-4), 50.1 (C-9), 51.3 (C-24), 56.0 (C-17), 56.8 (C-14), 71.8 (C-3), 121.8 (C-6), 129.3 (C-23), 138.4 (C-22), and 140.8 (C-5).

#### Tetracosane (5)

The fraction CC1-F_1_ (0.12 g) was precipitated to obtain compound **5**. Compound **5** was obtained as a white waxy solid (10.8 mg), and the data for its structural elucidation were as follows: UV (EtOH) λ_max_ nm (log ε): 202 (1.35); IR (ATR) ʋ_max_, cm^− 1^: 2915, 2849, 1473, 1463, 1262, 1021, 1096, 802, 729, and 719; HR-ESI-MS *m/z*: 338.3369 [M^+^] (calculated for C_24_H_50_, 338.3913 g/mol); ^1^H-NMR (CDCl_3_, 600 MHz) δ_H_ (ppm): 0.88 (6H, *m*, H-1, H-24), 1.27 (40H, *m*, H-3-H-22), and 1.32 (4H*, m*, H-2, H-23).

#### Margaric acid, (6)

The fraction CC1-F_10_ (1.84 g) was fractionated using Sephadex LH-20 column chromatography with 1% methanol in chloroform to yield ten fractions. The fourth fraction was then eluted using silica gel column chromatography with chloroform-ethyl acetate (4:1) to obtain ten more fractions. The fourth fraction was further purified using silica gel column chromatography with chloroform-ethyl acetate (3:1) to obtain compound **6**. Compound **6** was obtained as a white amorphous solid (9.0 mg), and the data for its structural elucidation were as follows: UV (EtOH) λ_max_ nm (log ε): 202 (2.47); IR (ATR) ʋ_max_, cm^− 1^: 2916, 2848, 1706, 1697, 1463, 1430, 1411, 1310, 1295, 1272, 1251, 1229, 1208, 1188, 939, and 720; EI-MS *m/z*: 269 [M-1]^+^ (calculated for C_17_H_34_O_2_, 270.0 g/mol); ^1^H-NMR (CDCl_3_, 600 MHz) δ_H_ (ppm): 0.89 (3H, *t, J =* 7.2, H-17), 1.26–1.32 (26H, *m,* H-4-H-16, *overlapped*), 1.65 (2H, *m,* H-3), and 2.35 (2H, *t, J* = 7.5 Hz, H-2); ^13^C-NMR (CDCl_3_, 150 MHz) δ_C_ (ppm): 14.2 (C-17), 22.7 (C-16), 24.7 (C-3), 29.1–31.9 (C-4-C-15), 34.1 (C-2), and 180.3 (C-1).

### Statistical analysis

Assay data obtained were subjected to one-way ANOVA with post-hoc Tukey’s multiple comparisons test using GraphPad Prism 5 software (La Jolla, CA, USA). The data are expressed as mean ± standard error of the mean (S.E.M.) with triplicate measurements (*n* = 3). The difference between means was determined at 95% confidence intervals, with *p* value < 0.05 considered as significantly different.

## Results

### In vitro xanthine oxidase inhibitory activity of *M. pumilum* varieties

Among the eighteen extracts assayed*,* five exhibited more than 50% xanthine oxidase inhibition, and their inhibitions were less than that of the positive control, allopurinol (99.82 ± 0.00%, IC_50_ = 0.24 ± 0.00 μg/mL). They were the dichloromethane extracts of var. *alata* (68.21 ± 2.50%, IC_50_ = 310.9 ± 8.25 μg/mL), var. *pumila* (85.77 ± 0.70%, IC_50_ = 161.6 ± 7.35 μg/mL), and var. *lanceolata* (74.33 ± 4.33%, IC_50_ = 233.1 ± 19.85 μg/mL) leaves, and the methanol extracts of var. *pumila* (80.97 ± 0.72%, IC_50_ = 175.1 ± 0.20 μg/mL) and var. *lanceolata* (67.52 ± 0.35%, IC_50_ = 185.3 ± 2.50 μg/mL) leaves (Tables 1 and 2). The dichloromethane extract of var. *pumila* leaves was considered to be more active than the other extracts because it had the highest percentage of xanthine oxidase inhibition and the lowest IC_50_ value. Thus, the extract was subjected to further fractionation processes that led to the isolation of six pure compounds.

**Table 1:** Percentages of xanthine oxidase inhibition of extracts of *Marantodes pumilum* varieties and allopurinol

Species	Plant part	Crude extracts	Yield (%)	Percentage of xanthine oxidase inhibition (%)^**a**^
*M. pumilum* var. *alata*	Roots	DCM	0.85	0.00
		MeOH	8.08	0.00
		H_2_O	6.00	0.00
	Leaves	DCM	1.80	68.21 ± 2.50
		MeOH	2.23	41.15 ± 4.31
		H_2_O	2.74	0.00
*M. pumilum* var. *pumila*	Roots	DCM	1.59	0.00
		MeOH	4.63	0.00
		H_2_O	2.58	0.00
	Leaves	DCM	1.38	85.77 ± 0.70
		MeOH	1.09	80.97 ± 0.72
		H_2_O	4.56	0.00
*M. pumilum* var. *lanceolata*	Roots	DCM	3.16	0.00
		MeOH	7.21	0.00
		H_2_O	5.16	0.00
	Leaves	DCM	3.30	74.33 ± 4.33
		MeOH	8.70	67.52 ± 0.35
		H_2_O	1.03	0.00
Allopurinol (positive control)				99.82 ± 0.00

Data are presented as mean ± S.E.M. of three replicates (*n* = 3)

^a^Percentage of xanthine oxidase inhibition of extracts and allopurinol were determined at concentration of 400 μg/mL and 100 μg/mL, respectively

**Table 2:** IC_50_ values of xanthine oxidase inhibition of selected extracts of *Marantodes pumilum* varieties and allopurinol

Species	Plant part	Crude extracts	Concentration (μg/mL)	Percentage of xanthine oxidase inhibition (%)	IC_**50**_ value of xanthine oxidase inhibition (μg/mL)^**a**^
*M. pumilum* var. *alata*	Leaves	DCM	25	28.08 ± 0.18	310.9 ± 8.25
			50	15.94 ± 0.00	
			100	3.99 ± 0.00	
			200	20.49 ± 0.22	
			400	68.21 ± 2.50	
*M. pumilum* var. *pumila*	Leaves	DCM	25	15.34 ± 0.25	161.6 ± 7.35
			50	14.31 ± 0.89	
			100	23.85 ± 0.05	
			200	65.28 ± 1.81	
			400	85.77 ± 0.70	
		MeOH	25	4.94 ± 0.29	175.1 ± 0.20
			50	9.86 ± 1.51	
			100	20.08 ± 0.05	
			200	60.73 ± 0.72	
			400	80.97 ± 0.72	
*M. pumilum* var. *lanceolata*	Leaves	DCM	25	26.90 ± 3.09	233.1 ± 19.85
			50	13.60 ± 2.93	
			100	28.89 ± 0.87	
			200	35.59 ± 1.40	
			400	74.33 ± 4.33	
		MeOH	25	19.15 ± 0.04	185.3 ± 2.50
			50	11.25 ± 1.21	
			100	37.98 ± 0.88	
			200	53.37 ± 0.37	
			400	67.52 ± 0.35	
Allopurinol (positive control)			0.0064	4.12 ± 0.00	0.24 ± 0.00
			0.032	17.81 ± 0.00	
			0.16	29.47 ± 0.00	
			0.8	93.66 ± 0.00	
			4	98.86 ± 0.00	
			20	99.01 ± 0.00	
			100	99.82 ± 0.00	

Data are presented as mean ± SEM. of three replicates (*n* = 3)

^a^IC_50_ values were obtained based on the percentage of xanthine oxidase inhibition of extracts and allopurinol at different concentrations using the Graphpad Prism 5 software (La Jolla, CA, USA)

### Structural elucidation of compounds isolated from the dichloromethane extract of *M. pumilum* var. *pumila* leaves and their xanthine oxidase inhibitory activity

**Fig. 2:**
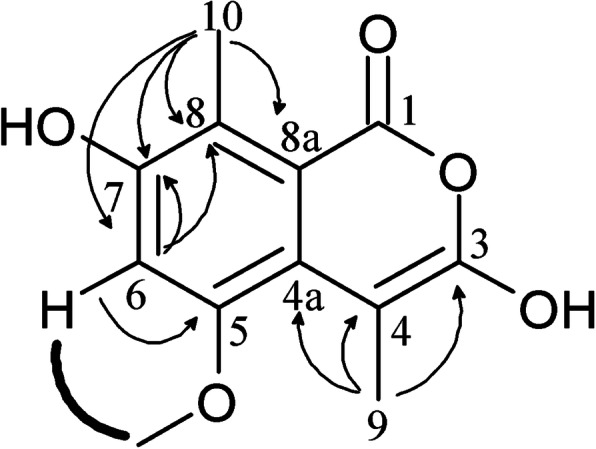
HMBC (H → C) and COSY (H▬H) correlations for compound **1** obtained using NMR spectrometric technique

Compound **1** was obtained as a white amorphous powder from the dichloromethane fraction, and its molecular formula was established as 7 degrees of unsaturation. Its UV spectrum showed maximum absorption at 275 nm. Its ATR-FTIR spectrum showed strong absorption at 3256 (O-H stretching), 1734 (C=O), 1473–1424 (C-H bending), and 1256 (C-O stretching) cm^− 1^. Its ^1^H-NMR spectrum (CDCl_3_, 600 MHz) showed methyl protons at δ 2.45 (3H, H-9) and δ 2.65 (3H, *s,* H-10), a methoxy proton at δ 3.89 (3H, *s,* H-5), and an aromatic proton at δ 6.91 (*s*). Its ^13^C-NMR spectrum (CDCl_3_, 150 MHz) showed 12 carbons with signals indicating the presence of one carbonyl carbon at δ 162.7 (C-1), one methoxy carbon at δ 153.1 (C-5), and two methyl carbons at δ 18.8 (C-11) and δ 15.2 (C-12). Its ^1^H-^1^H COSY spectrum displayed the correlation between aromatic protons at δ 6.91 (*s*) and methoxy protons at δ 3.89, thus revealing the location of the aromatic proton group at the C-6 position. Its HSQC spectrum showed correlations between methoxy protons at δ 3.89 and δ 56.6 (C-5), an aromatic proton at δ 6.9 and δ 101.8 (C-6), and two methyl protons at δ 2.45 and δ 2.65 and δ 18.8 (C-9) and δ 15.2 (C-10), respectively. In its HMBC spectrum, the linkage of two methyl protons was established by the cross peaks between H-9 (δ 2.45) and C-4 (δ139.9, ^2^*J*), C-4a (δ110.2, ^3^*J*), and C-3 (δ157.1, ^3^*J*), and between H-10 (δ 2.65) and C-7 (δ 142.5, ^3^*J*), C-8 (δ 119.8, ^2^*J*), and C-8a (δ 131.1, ^3^*J*). This correlation confirmed the position of methyl protons in compound **1**. The spectrum also showed the correlation between methoxy protons at δ 3.89 and C-5 (δ 153.1, ^2^*J*), and the correlation between aromatic protons (δ 6.91) and C-5 (δ 153.1, ^2^*J*), C-7 (δ 142.5, ^2^*J*), and C-8 (δ 119.8, ^3^*J*). Therefore, based on the data above the structure of compound **1** was determined as 3,7-dihydroxy-5-methoxy-4,8-dimethyl-isocoumarin (Fig. 2). All ^1^H-NMR and ^13^C-NMR data of compound **1** are shown in Table 3.

**Table 3:** ^1^H- and ^13^C-NMR spectra data for compound **1**

No	δ_C_ (ppm)	δ_H_ (ppm)
1	162.7	**–**
3	157.1	**–**
4	139.9	**–**
4a	110.2	**–**
5	153.1	**–**
OCH_3_	56.6	3.89, *s*
6	101.8	6.91, *s*
7	142.5	–
OH	–	–
8	119.8	–
8a	131.1	
9	18.8	2.45, *s*
10	15.2	2.65, s

**Fig. 3:**
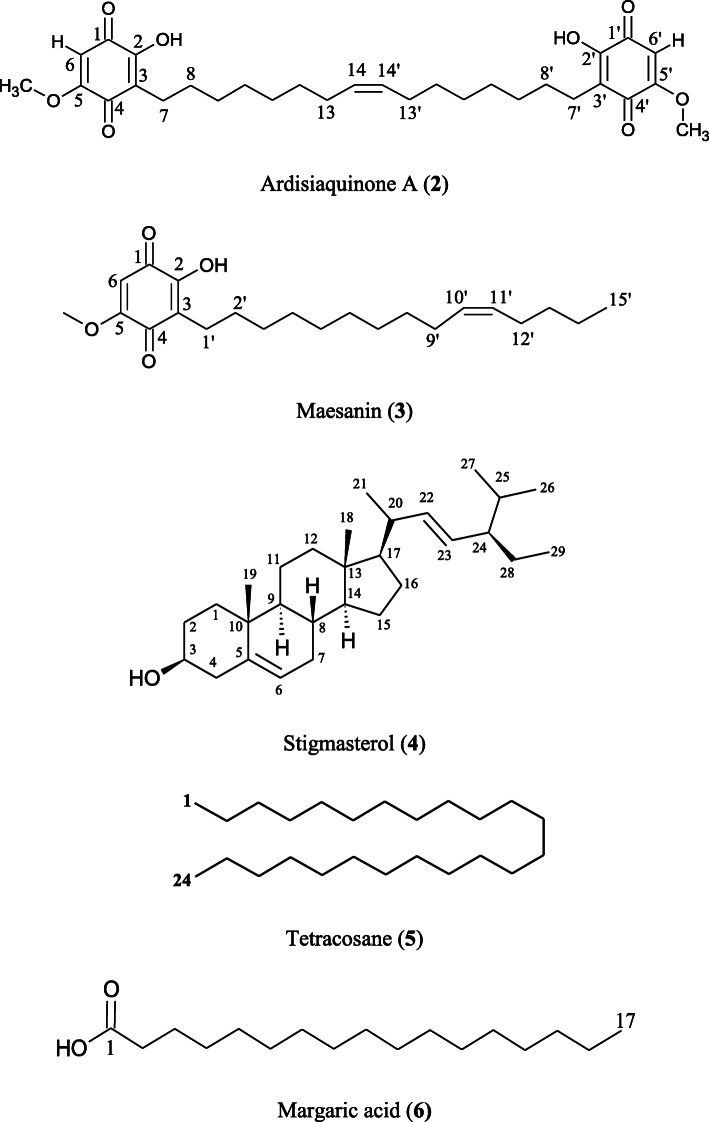
Chemical structures of compounds **2**–**6** elucidated using UV, IR, MS, and NMR spectroscopic techniques

The other five known compounds were identified as ardisiaquinone A (**2**) [41], maesanin (**3**) [42], stigmasterol (**4**) [43], tetracosane (**5**) [44], and margaric acid (**6**) [45] (Fig. 3) by comparing MS and NMR data with those reported in the literature.

Of the six compounds, only 3,7-dihydroxy-5-methoxy-4,8-dimethyl-isocoumarin (**1**) and ardisiaquinone A (**2**) exhibited more than 50% xanthine oxidase inhibition (Table 4), with the former (**1**) being more potent than the latter (**2**). The former (**1**) had an IC_50_ value of 0.66 ± 0.01 μg/mL, which is comparable (*p* > 0.05) with that of allopurinol (IC_50_ = 0.24 ± 0.00 μg/mL) (Table 5).

**Table 4:** Percentages of xanthine oxidase inhibition of isolated compounds and allopurinol

Compound	Percentage of xanthine oxidase inhibition (%)^**a**^
**1**	98.46 ± 0.37
**2**	91.54 ± 0.08
**3**	0.00
**4**	0.00
**5**	0.00
**6**	0.00
Allopurinol (positive control)	99.82 ± 0.00

Data are presented as mean ± S.E.M. of three replicates (*n* = 3)

^a^Percentage of xanthine oxidase inhibition of compounds and allopurinol were determined at a concentration of 100 μg/mL

**Table 5:** IC_50_ values of xanthine oxidase inhibition of compounds **1** and **2** compared to allopurinol

Compound	Concentration (μg/mL)	Percentage of xanthine oxidase inhibition (%)	IC_**50**_ value of xanthine oxidase inhibition (μg/mL)^**a**^
**1**	0.39	39.57 ± 0.68	0.66 ± 0.01 ^b^
	0.78	54.14 ± 0.34	
	1.56	66.49 ± 0.05	
	3.13	76.17 ± 0.52	
	6.25	86.02 ± 0.14	
	12.5	91.75 ± 0.22	
	25	95.04 ± 0.86	
	50	96.91 ± 0.06	
	100	98.46 ± 0.37	
**2**	6.25	9.11 ± 0.28	31.2 ± 1.28
	12.5	14.54 ± 0.45	
	25	36.31 ± 0.08	
	50	74.08 ± 0.50	
	100	91.54 ± 0.08	
Allopurinol (positive control)	0.0064	4.12 ± 0.00	0.24 ± 0.00
	0.032	17.81 ± 0.00	
	0.16	29.47 ± 0.00	
	0.8	93.66 ± 0.00	
	4	98.86 ± 0.00	
	20	99.01 ± 0.00	
	100	99.82 ± 0.00	

Data are presented as mean ± S.E.M. of three replicates (*n* = 3)

^a^IC_50_ values were obtained based on the percentage of xanthine oxidase inhibition of compounds and allopurinol at different concentrations using the Graphpad Prism 5 software (La Jolla, CA, USA)

^b^Not significantly different compared to allopurinol (*p* > 0.05), analyzed using one-way ANOVA followed by Tukey’s multiple comparisons test

## Discussion

The extract of *M. pumilum* var. *pumila* leaves inhibited xanthine oxidase in vitro. The findings of this study support the previous report [39] in which ethanol (80%) *M. pumilum* var. *pumila* leaf extract showed anti-hyperuricemic effect by inhibiting hepatic xanthine oxidase and reducing serum uric acid levels in hyperuricemic-induced male Sprague-Dawley rats 14 days after treatment with 200 mg/kg extract.

In this study, a new compound (3,7-dihydroxy-5-methoxy-4,8-dimethyl-isocoumarin) was isolated from the dichloromethane extract of *M. pumilum* var. *pumila* leaves, which was found to be the most active extract (IC_50_ = 161.6 ± 7.35 μg/mL). The compound had an IC_50_ value (0.66 ± 0.01 μg/mL) that was comparable to that of allopurinol (IC_50_ = 0.24 ± 0.00 μg/mL) and could be a potential xanthine oxidase inhibitor. A study by Lin et al. [46] demonstrated competitive inhibition of selected coumarins (e.g., coumarin, 4-hydroxycoumarin, 7-hydroxycoumarin, esculetin, scopoletin, dihydrocoumarin, and 7-hydroxy-4-methylesculetin) against xanthine oxidase. Esculetin was found to be the most potent inhibitor through substrate binding blockade. It was suggested that the two hydroxyl moieties on its benzene ring contributed to its activity by forming hydrogen bonds with the active site of xanthine oxidase. Therefore, the presence of two hydroxyl groups in the structure of 3,7-dihydroxy-5-methoxy-4,8-dimethyl-isocoumarin (**1**) could explain the basis of its xanthine oxidase inhibitory activity. Another study also reported that the xanthine oxidase inhibitory activity of 5,7-dihydroxy-3-(3-hydroxyphenyl) coumarin was 7-fold better than that of allopurinol [47]. The low activity of ardisiaquinone A (**2**) and lack of activity of the other isolated compounds (**3**–**6**) obtained in this study could be explained by the difference in molecular structure that influences the stability of hydrophilic and hydrophobic characteristics on the xanthine oxidase active binding site [48].

There are several reviews on the anti-hyperuricemic effects of foods [49], Chinese herbs [50], and natural products [51]. Hyperuricemia has been linked with cardiovascular disease, hypertension, diabetes, obesity, chronic kidney disease, and many other diseases [52, 53]. Its prevalence in the female population and post-menopausal women has been reported [54–56]. The data from the Third National Health and Nutrition Examination Survey showed that menopause was associated with higher serum uric acid levels and postmenopausal hormone replacement was associated with lower serum uric acid levels, suggesting that estrogen plays a key role in protecting women from hyperuricemia and gout [57]. Several publications have reported on the potential use of *M. pumilum* extract to alleviate postmenopausal conditions due to estrogenic properties [58–60], hypercholesterolemia [61], and hypertension [62]. Thus, the extract of *M. pumilum* var. *pumilum* could be beneficial in preventing or treating hyperuricemic-related diseases, while 3,7-dihydroxy-5-methoxy-4,8-dimethyl-isocoumarin (**1**) could be used as an analytical marker to standardize the extract and formulated herbal products. Standardization by simultaneous quantification of xanthine oxidase inhibitors from *Zanthoxylum armatum* fruits using high-performance liquid chromatography with a photometric diode array detector (HPLC-PDA) has been reported [63].

## Conclusions

In the present study, three varieties of *M. pumilum* were investigated based on their ethnomedical uses and biological activities. The study identified a new isocoumarin compound, 3,7-dihydroxy-5-methoxy-4,8-dimethyl-isocoumarin (**1**), from the dichloromethane extract of *M. pumilum* var. *pumila* leaves. The compound was the most active xanthine oxidase inhibitor and had an IC_50_ value (0.66 ± 0.01 μg/mL) that was comparable with that of allopurinol (IC_50_ = 0.24 ± 0.00 μg/mL). Therefore, *M. pumilum* var. *pumila* leaves could potentially be a source of new natural xanthine oxidase inhibitors. However, in vivo studies are required to establish its efficacy and safety.

## Data Availability

All relevant data regarding the study is included in this article and any supplementary data is available from the corresponding author upon request.

## Abbreviations

IC_50_: The half maximal inhibitory concentration
HPTLC: High performance thin later chromatography
HPLC: High performance liquid chromatography
ATR-FTIR: Attenuated total reflectance-fourier transform infrared spectroscopy
XO: Xanthine oxidase
VLC: Vacuum liquid chromatography
CC: Column chromatography
TLC: Thin layer chromatography
UV: Ultraviolet spectrophotometry
IR: Infrared spectrophotometry
NMR: Nuclear magnetic resonance spectroscopy
HR-ESI-MS: High-resolution electrospray ionization mass spectrometry
EI-MS: Electron ionization mass spectrometry
DCM: Dichloromethane
MeOH: Methanol
CHCl_3_: Chloroform
DMSO: Dimethyl sulfoxide
EtOAc: Ethyl acetate
EtOH: Ethanol
HCl: Hydrochloric acid
NaOH: Sodium hydroxide
KH_2_PO_4_: Potassium dihydrogen phosphate
UKMB: Herbarium of Universiti Kebangsaan Malaysia in Bangi
λ_max_: Wavelength of maximum absorbance
log ε: Log of molar absorption coefficient
ʋ_max_: Maximum frequency
*m/z*: Mass-to-charge ratio
^1^H-NMR: Proton nuclear magnetic resonance
CDCl_3_: Deuterated chloroform
δ_H_: Proton chemical shift
*s*: Singlet
^13^C-NMR: Carbon-13 nuclear magnetic resonance
δ_C_: Carbon chemical shift
*m*: Multiplet
*d*: Doublet
*J*: Coupling constant
*br*: Broad
CD_3_OD: Deuterated methanol
*t*: Triplet
*dd*: Double doublet
S.E.M.: Standard error of the mean
*n*: Number
ANOVA: Analysis of variance
*p*: Probability
COSY: Correlated spectroscopy
HSQC: Heteronuclear single quantum coherence spectroscopy
HMBC: Heteronuclear multiple bond correlation spectroscopy
HPLC-PDA: High performance liquid chromatography-photometric diode array
